# Ontogenesis of NADPH-diaphorase positive neurons in guinea pig neocortex

**DOI:** 10.3389/fnana.2015.00011

**Published:** 2015-02-16

**Authors:** Chao Liu, Yan Yang, Xia Hu, Jian-Ming Li, Xue-Mei Zhang, Yan Cai, Zhiyuan Li, Xiao-Xin Yan

**Affiliations:** ^1^Department of Anatomy and Neurobiology, Central South University School of Basic Medical ScienceChangsha, China; ^2^Department of Neurology, The First Hospital of ChangshaChangsha, China; ^3^School of Nursing, Xiangtan Vocational and Technical CollegeXiangtan, China; ^4^Department of Neurology, The Second Affiliated Hospital, Harbin Medical UniversityHarbin, China

**Keywords:** corticogenesis, GABAergic, interneuron, neuronal development, nitric oxide

## Abstract

In mammalian cerebrum there exist two distinct types of interneurons expressing nitric oxide synthase (NOS). Type I neurons are large in size and exhibit heavy nicotinamide adenine dinucleotide phosphate diaphorase (NADPH-d) histochemical reaction, while type II cells are small with light NADPH-d reactivity. The time of origin of these cortical neurons relative to corticogenesis remains largely unclear among mammals. Here we explored this issue in guinea pigs using cell birth-dating and double-labeling methods. Bromodeoxyuridine (BrdU) pulse-chasing (2 doses at 50 mg/kg, 12 h apart) was given to time-pregnant mothers, followed by quantification of NADPH-d/BrdU colocalization in the parietal and temporal neocortex in offspring at postnatal day 0 (P0), P30 and P60. Type I neurons were partially colabeled with BrdU at P0, P30 and P60 following pulse-chasing at embryonic day 21 (E21), E28 and E35, varied from 2–11.3% of total population of these neurons for the three time groups. Type II neurons were partially colabeled for BrdU following pulse-chasing at E21, E28, E35 and E42 at P0 (8.6%–16.5% of total population for individual time groups). At P60, type II neurons were found to co-express BrdU (4.8–11.3% of total population for individual time groups) following pulse-chasing at E21, E28, E35, E42, E49, E56 and E60/61. These results indicate that in guinea pigs type I neurons are generated during early corticogenesis, whereas type II cells are produced over a wide prenatal time window persisting until birth. The data also suggest that type II nitrinergic neurons may undergo a period of development/differentiation, for over 1 month, before being NADPH-d reactive.

## Introduction

Nitric oxide (NO) plays important roles in the nervous system via modulation of neurotransmission, synaptic plasticity and coupling of vasodilatation with neuronal activity (Belvisi et al., 1995; Salemme et al., 1996; Guix et al., 2005; Sunico et al., 2005; Melikian et al., 2009; Toda et al., 2009; Hardingham et al., 2013). Normal NO activity is required for a broad range of physiological processes including neurogenesis (Gibbs, 2003; Matarredona et al., 2005), learning and memory (Wultsch et al., 2007; Majlessi et al., 2008; Paul and Ekambaram, 2011), sleep (Cudeiro et al., 2000; Gautier-Sauvigné et al., 2005; Gerashchenko et al., 2008; Greene, 2013) and neuroendocrine (Ceccatelli, 1997; Riedel, 2000; Garrel et al., 2002; Givalois et al., 2002). Aberrant NO signaling may be involved in some pathophysiological or disease conditions such as brain aging (McCann et al., 2005), neural stress and inflammation (Cherian et al., 2004; Gądek-Michalska et al., 2013), neuropathological pain (Zimmermann, 2001), neurodegenerative diseases (Togo et al., 2004; Aquilano et al., 2008) and psychiatric disorders (McLeod et al., 2001; Reif et al., 2006; Colvin and Kwan, 2014; Weber et al., 2014).

NO is produced by nitric oxide synthase (NOS) that exists as neuronal (nNOS), endothelial (eNOS) and inducible (iNOS) isoforms. In the brain nNOS and eNOS are expressed constitutively and can be conventionally visualized with nicotinamide adenine dinucleotide phosphate-diaphorase (NADPH-d) histochemistry (Dawson et al., 1991; Hope et al., 1991). Two types of NADPH-d positive neurons, both being interneurons, were proposed based on morphological and neurochemical properties of the cells in nonhuman primate cerebral cortex, with type I neurons being large in size with heavy NADPH-d reactivity, while type II cells small with light histochemical reaction (Yan et al., 1996a). These two types of cells are also apparently present in guinea pig, rabbit, cat and human cerebral cortex (Lüth et al., 1994; Yan et al., 1996b; Yan and Garey, 1997a; Estrada and DeFelipe, 1998; Judas et al., 1999; Garbossa et al., 2005; Cruz-Rizzolo et al., 2006; D’Alessio et al., 2007). In mice and rats, type I cells are consistently described in the cerebral cortex in many reports (Bredt et al., 1990; Leigh et al., 1990; Dawson et al., 1991; Vincent and Kimura, 1992; Aoki et al., 1993; Valtschanoff et al., 1993; Rodrigo et al., 1994; Yan et al., 1994; Nogueira-Campos et al., 2012), while a few studies also demonstrate type II cells in these small rodents (Freire et al., 2012; Magno et al., 2012). Colocalization studies with early cell lineage markers have established that in mice the majority of type I neurons arise from the medial ganglionic eminence (MGE), whereas type II cells have multiple origins including the MGE, lateral/caudal ganglionic eminences (LGE/CGE) and the preoptic area (Jaglin et al., 2012; Magno et al., 2012).

Guinea pig brain develops via a relatively longer period of prenatal morphogenesis and has a greater encephalization quotient in the adult, relative to mice and rats (Rice et al., 1985; Herculano-Houzel, 2007). Also unlike the latter small rodents, but similar to cats and monkeys, type II NADPH-d and doublecortin expressing cells are prominent in adult guinea pigs neocortex (Yan and Garey, 1997a; Cai et al., 2009; Xiong et al., 2008; Bonfanti and Nacher, 2012). Thus, guinea pigs might be suited for addressing certain phylogenetically-related issues about mammalian cortical neurogenesis, such as interneuron formation (Cahalane et al., 2014; Hladnik et al., 2014). Here we characterized the ontogenesis of NADPH-d neurons in guinea pig neocortex using 5-bromodeoxyuridine (BrdU) birth-dating and double-labeling histological methods.

## Materials and methods

### Animals and prenatal BrdU administration

Animal use was in accordance with the *National Institute of Health Guide for the Care and Use of Laboratory Animals*. All experimental procedures in the present study were approved by the Ethics Committee of Central South University Xiangya School of Medicine for animal care and use. Efforts were also made to minimize stress and pain, and to avoid unnecessary use of experimental animals.

Mating pairs of Hartley guinea pigs aged 4–6 months were purchased from the animal center of Xiangya School of Medicine. Time-pregnant mothers received two doses of 5-BrdU (B5002, Sigma-Aldrich, St Louis, MO, USA) injection (50 mg/kg, 12 h apart, i.p.) at embryonic day 14 (E14), E21, E28, E35, E42, E49, E56 and E60-61 for the expected offspring. This prenatal BrdU chasing procedure was applied to 3 mothers for each of the above injection time points. Pregnant guinea pigs gave live-birth pups of 2–4. Brains of the postnatal guinea pigs were examined at the day of birth, defined as postnatal day 0 (P0), or following development to 1 (P30) and 2 (P60) months of age. For each time point, 3–4 animals were studied at P0, 2–3 animals at P30 and 3–4 animals at P60. Postnatal animals were coded according to the embryonic day receiving BrdU injection and the day of brain perfusion (e.g., E14-P0). BrdU injections were also applied to pregnant animals at E14 (*n* = 3), which resulted in aborted pregnancy with no offspring available for study.

### Tissue preparation

Animals were anesthetized with sodium pentobarbital (100 mg/kg, i.p.), followed by transcardiac perfusion with 4% paraformaldehyde in 0.01 M phosphate-buffered saline (pH 7.4, PBS). Brains were dissected out, postfixed overnight and immersed in 30% sucrose for cryoprotection. The forebrain was cut at the frontal plane in a cryostat at a thickness of 30 μm, with 24 sets of sections collected serially in PBS in cell culture plates. At least one set of sections from each brain was processed for histology (Nissl stain), NADPH-d histochemistry, BrdU immunohistochemistry and NADPH-d/BrdU double-stain, respectively, either immediately after sectioning or following a period of storage of the sections in a cryoprotectant at 20°C.

### NADPH-diaphorase histochemistry

Two sets of sections from each brain were processed in parallel by incubation in 0.05 M Tris-HCl buffered saline (pH 8.0, TBS) containing 0.3% Triton X-100, 1 mM nicotinamide adenine dinucleotide phosphate diaphorase (β-NADPH-d, N7505, Sigma-Aldrich, St Louis, MO, USA), 0.8 mM nitroblue tetrazolium (NBT, N6639, Sigma-Aldrich) and 5% dimethyl sulfoxide for 45 min at 37°C (Yan et al., 1994). The reaction was stopped by rinsing sections with PBS in room temperature. The sections were then mounted for histological examination or processed further for BrdU immunolabeling using DAB as a chromogen (see below).

### BrdU immunohistochemistry

A set of new sections and selected sections stained with NADPH-d histochemistry described above were simultaneously processed for BrdU immunolabeling with the DAB-peroxidase method. Sections were pre-treated in 1 × SSC and 50% formamide for 1 h at 65°C, in 2N HCl for 30 min at 37°C, and in PBS containing 1% H_2_O_2_, 5% normal rabbit serum and 0.3% Triton X-100 for 45 min. Sections were next incubated overnight at 4°C with rat anti-BrdU (AbD Serotec, Raleigh, NC, USA, MCA2060, 1:2000) diluted in PBS containing 5% rabbit serum, reacted with biotinylated rabbit anti-rat IgG at 1:400 for 2 h, and subsequently with the ABC reagents (1:400) (Vector Laboratories, Burlingame, CA, USA) for an additional hour. Immunoreactivity was visualized in 0.003% H_2_O_2_ and 0.05% diaminobenzidine.

### Imaging, cell count and data processing

An Olympus microscope (BX53) equipped with a digital imaging system (CellSens Standard, Olympus, Japan) was used for tissue observation and imaging. Sections from the levels of the striatum to mid-hippocampus were used for morphometry of NADPH-d and BrdU/NADPH-d colocalized neurons. Images were taken from 5 equally-spaced sections in each brain at 20×, continuously along the pia for ~3 mm distance (6 image wide) above and below (~1 mm away from the sulcus, where the cortex become flat) the lateral sulcus, respectively, covering both the cortex and white matter regions. The above region in an adjacent Nissl stain section was also imaged at 10× for laminar reference including areal measurement. The images from the same section were montaged, with cortical laminar borders in BrdU/NADPH-d preparation marked by referring to the adjacent Nissl stain images. Cell count was carried out on-screen at high resolution in the montaged BrdU/NADPH-d images, while laminar areas were obtained in the Nissl stain montages using Image I. Layer 1 derives from the embryonic marginal zone (MZ), whereas layers 2–6 arise from the cortical plate (CP). Considering this corticogenesis feature as well as the differential laminar distribution of type I and type II nitrigeneric neurons in adult guinea pigs (Yan and Garey, 1997a), cell identification and count were carried out according to four laminar sectors, layer 1, layers 2–4, layers 5/6 and the white matter. Cells were classified and marked, with the numbers of NADPH-d labeled and BrdU/NADPH-d colabeled neurons in each laminar sector recorded. The percentages of double-labeled relative to total NADPH-d cells (type I and type II respectively) were then calculated for each animal. In a given animal, at least 300 type I and 1000 type II NADPH-d positive neurons (with and without BrdU colocalization) were counted. The areal densities of NADPH-d neurons were calculated using the sums of cells and the areas measured for corresponding laminar sectors.

### Statistical testing and figure preparation

Means of density or percentage values were calculated for each animal using the data from the above systemically sampled sections and laminar regions, and entered into the Prism spreadsheet (Prism GraphPad 4.1, San Diego, CA, USA) for graph preparation. The means and standard derivations (SD) were also calculated for individual groups using this set of data. Kruskal-Wallis Test with Dunn’s Multiple Comparison was used for statistical analysis. The minimal significant level of difference was set at *p* < 0.05. Figures were assembled with Photoshop 7.1.

## Results

### Cortical NADPH-d neurons in neonatal and young adult guinea pigs

**Figure 1:**
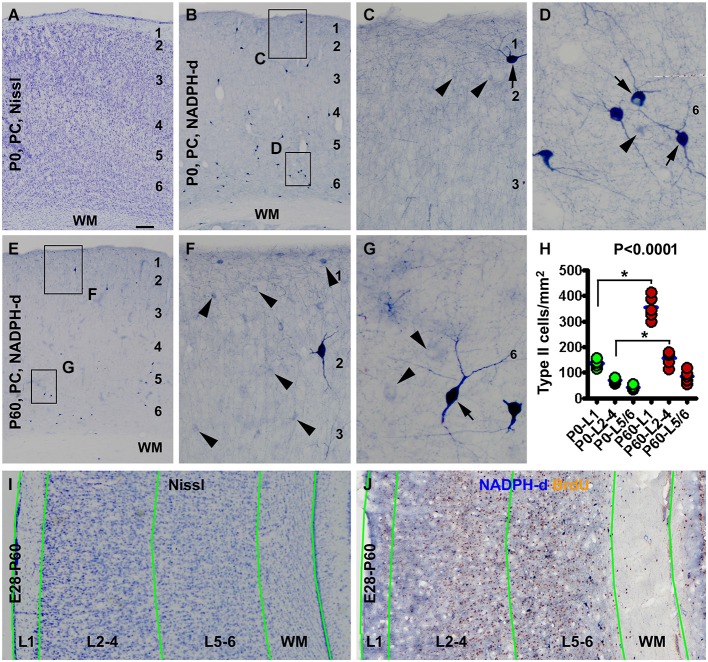
**Laminar architecture, nicotinamide adenine dinucleotide phosphate-diaphorase (NADPH-d) positive neurons in the neocortex of neonatal and 2 month-old (postnatal day 60, P60) guinea pigs, and methodological illustration for lamina-based cell count**. Panel **(A)** shows a Nissl stain image of the parietal neocortex (PC) around the border of the primary motor (left portion) and somatosensory (right portion) cortex from a newborn (P0). Layers 1 to 6 are identifiable above the white matter (WM). Panel **(B)** is a low magnification image of NADPH-d neurons in the somatosensory cortex of a newborn, with the framed areas enlarged as **(C)** and **(D)**. Panel **(E)** is a low power view of NADPH-d reactivity in the somatosensory cortex in a P60 animal, with the framed areas enlarged as **(F)** and **(G)**. Examples of type I and type II NADPH-d neurons are pointed by arrows and arrowheads, respectively. The densities of type II NADPH-d neurons in layer 1 (L1), layers 2–4 (L2-4) and layers 5 and 6 (L5/6) in the parietotemporal neocortex are increased in the P60 (*n* = 5) relative to P0 (*n* = 5) groups (calculated based on the cell count data obtained from NADPH-d/BrdU dual staining preparations) **(H)**. For counting NADPH-d positive and NADPH-d/BrdU colabeled neurons, the sampled parietal and temporal cortical regions are divided into L1, L2-4, L5/6 and white matter (WM). Borders of the laminar sectors are created in the montaged Nissl stain image of an adjacent section **(I)**, which were copied as a template and then pasted on the montaged dual staining image **(J)**. The size of the template is adjusted proportionally by aligning the pial and ventricular lines as closely as possible. NADPH-d neurons in each laminar sector are classified and counted on screen at high resolution. Scale bar = 100 μm in **(A)** applying to **(B,E)**, equivalent to 50 μm for **(I,J)**, 20 μm for **(C,F)** and 10 μm for **(D,G)**.

Details of corticogenesis, including the establishment of cortical layers, in guinea pigs still remain largely uncharacterized (to our knowledge). However, a few previous studies have indicated that the basic lamination of the neocortex is readily established at birth (Schüz, 1981; Rice et al., 1985). Here we briefly note that the 6 layered basic architecture of the cerebral neocortex as seen in Nissl stain was established in the newborn (Figure 1A), as compared to young adults (Figure 1I).

Consistent with an early report (Yan and Garey, 1997a), we observed type I and type II NADPH-d neurons in guinea pig cerebral cortex in the present study, with both types readily present in the newborns (Figures 1B–D). The laminar distribution pattern of type I cells were comparable at the three postnatal time points examined, localizing primarily to layers 5 and 6 and the subcortical white matter, while a few of them scattered in the superficial layers, including layer 1 (Figures 1B–G). Also, there was no significant difference in the numerical density of these cells between the three age groups as quantified over layer I, layers 2–4 and layers 5/6 and subcortical white matter (data not shown).

Type II cells were distributed over layers 1–6 and rare in the white matter in the postnatal guinea pigs, denser in layers 1–4 than 5/6 in all postnatal animals. The numerical density of type II cells appeared to be increased at P60 relative to P0 (Figures 1C,F). Using the cell count data from the NADPH-d/BrdU preparations, the mean areal densities of type II cells at P0 (*n* = 5) were estimated to be 136.6 ± 15.4, 70.5 ± 9.8 and 42.2 ± 8.1 per square mm in layers 1, 2–4 and 5/6, respectively, while those at P60 (*n* = 5) were estimated to be 354.7 ± 45.9, 156.9 ± 28.1 and 85.4 ± 23.9 per square mm in layers 1, 2–4 and 5/6, respectively. The medians of the densities were significant different (*P* < 0.0001, Kruskal-Wallis test), with a significant increase of density in layer 1 and layers 2–4 at P60 relative to P0 (Figure 1H).

### Laminar distribution of BrdU/NADPH-d colabeled neurons in neonatal guinea pigs

Mother guinea pigs give birth following a pregnancy of approximately 9 weeks. In order to determine the lamination pattern of NADPH-d positive neurons during corticogenesis, we gave BrdU injection to pregnant guinea pigs at weekly intervals from E21 to just before birth (E60/61) for the expected offspring, and attempted to quantify the distribution of BrdU/NADPH-d colabeled cells against cortical laminar portions (Figures 1I,J).

**Figure 2:**
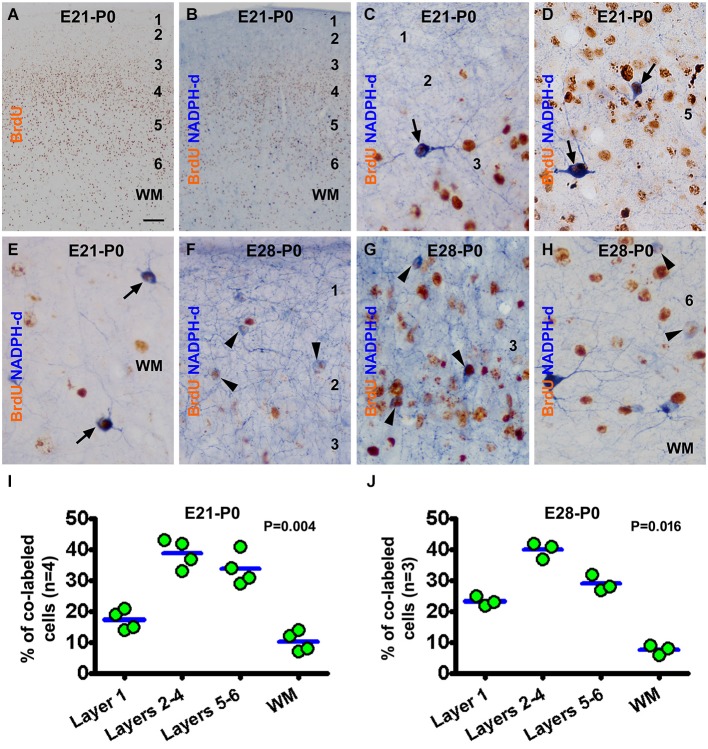
**Representative images and quantitative laminar analyses showing bromodeoxyuridine (BrdU) labeling and colocalization in NADPH-d neurons in neonatal guinea pig neocortex following pulse-chasing at embryonic day 21 (E21) and E28**. Panel **(A)** shows the distribution of BrdU immunoreactive nuclei largely in the middle layers of the neocortex in the E21-P0 group. Panel **(B)** illustrates a low power view of BrdU and NADPH-d dual staining. The remaining panels are high power views of BrdU/NADPH-d colocalized neurons in different cortical layers as marked, from the E21-P0 **(C–E)** and E28-P0 **(F–H)** groups. Examples of double labeled type I and type II cells are pointed by arrows and arrowheads, respectively. Dot graphs **(I,J)** show the laminar distribution of colabeled type I and type II neurons among the groups, with each dot representing the mean of an individual animal. The colabeled cells are present differentially (*P* = 0.004 by Kruskal-Wallis) between the laminar sectors. Arab numbers: cortical layers, WM: white matter. Scale bar = 100 μm in **(A)** applying to **(B)**, equivalent to 10 μm for other image panels.

In the newborns received BrdU on E21 (the E21-P0 group), BrdU immunoreactive cells were distributed mostly in the middle portion of the gray matter, i.e., layers 4/5, in the neocortex. A smaller amount of labeling occurred over layer 6 and the white matter, while layers 1–3 contained the least amount of BrdU immunoreactivity (Figures 2A,B). BrdU/NADPH-d colabeled neurons, evidently the type I cells but some type II cells as well, were observed across the cortex and white matter (Figures 2C–E). A similar pattern in the distribution of BrdU single labeling was seen in the neocortex in neonatal guinea pigs received BrdU on E28 (E28-P0) (image not shown). Also, both type I and type II cells were partially colocalized with BrdU in the E28-P0 group (Figures 2F–H). Quantitative data for the E21-P0 group indicated that 17.3 ± 2.96% of the colabeled cells occurred in layer 1, 38.8 ± 4.0% in layers 2–4, 33.8 ± 4.5% in layers 5/6, and 10.3 ± 2.9% in the white matter, with an overall difference among the laminar portions (*P* = 0.004) (Figure 2I). The cell count data from the E28-P0 group showed that 23.3 ± 1.5% of the colabeled cells occurred in layer 1, 40.0 ± 2.2% in layers 2–4, 29.0 ± 2.2% in layers 5/6, and 7.7 ± 1.3% in the white matter, with an differential laminar localization (*P* = 0.016) (Figure 2J).

**Figure 3:**
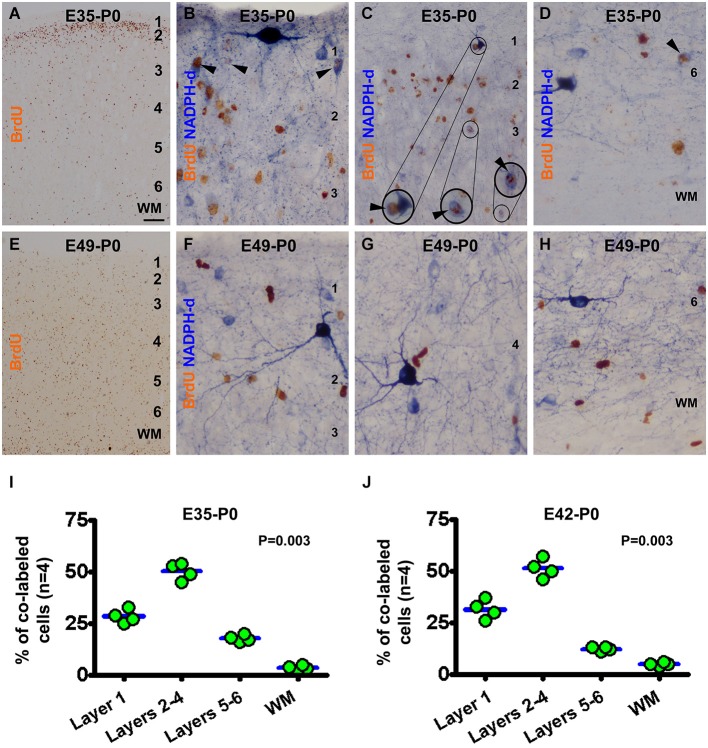
**Representative images and laminar analyses illustrating bromodeoxyuridine (BrdU) and BrdU/NADPH-d labeling in neonatal guinea pig neocortex following pulse-chasing at embryonic day 35 (E35, A–D), E42 (J, images not shown) and E49 (E–H)**. BrdU immunoreactive cells are predominately localized to layer 2 in the E35-P0 group **(A)**, but occur across the cortex without apparent laminar preference in the E49-P0 group **(E)**. BrdU/NADPH-d double-labeled neurons (pointed by arrowheads and enlargements in B-D) are differentially distributed among the laminar sectors (*P* = 0.004), mostly dense in layers 2–4 for both the E35-P0 **(I)** and the E42-P0 **(J)** groups. No BrdU/NADPH-d double-labeled neurons are found in the E49-P0 group **(E–H)**. Scale bar = 100 μm in **(A)** applying to **(E)**, equivalent to 20 μm for **(C)** and 10 μm for the remaining image panels.

In the newborns received BrdU on E35 (E35-P0), BrdU immunoreactive neurons were predominantly localized to layer 2 (Figure 3A). BrdU/NADPH-d colabeled neurons were found in the gray matter but rarely in the white matter (Figures 3B–D), with the great majority being type II cells (to be addressed quantitatively later). Quantitative data for this group revealed that 28.5 ± 3.0% of the colabeled cells occurred in layer 1, 50.3 ± 3.6% in layers 2–4, 17.8 ± 1.5% in layers 5/6, and 3.5 ± 1.1% in the white matter, exhibiting an overall difference between the laminar sectors (*P* = 0.003) (Figure 3I). In the E42-P0 group, BrdU immunolabeled cells were distributed across the entire cortical gray matter without apparent laminar preference (images not shown, similar to Figure 3E). Some type II cells, but none of type I cells, were found to colocalize with BrdU (images not shown). Cell count data revealed that 31.5 ± 4.0% of the colabeled cells occurred in layer 1, 51.3 ± 3.9% in layers 2–4, 12.38 ± 0.8% in layers 5/6, and 5.0 ± 0.7% in the white matter, with an differential laminar pattern (*P* = 0.003) (Figure 3J).

In the neonatal guinea pigs subjected to BrdU pulse-chasing on E49 (E49-P0 group) (Figures 3E–H), E56 (E56-P0 group, image not shown) and E60/61(E60/61-P0 group, image not shown), BrdU immunoreactive cells were scattered across the gray matter without apparent laminar preference (Figure 3E). No NADPH-d reactive neurons were found to co-express BrdU in the E49-P0 (Figures 3F–H), E56-P0 or E60/61-P0 groups (images not shown).

### Laminar distribution of BrdU/NADPH-d colabeled neurons in 2 month-old guinea pigs

**Figure 4:**
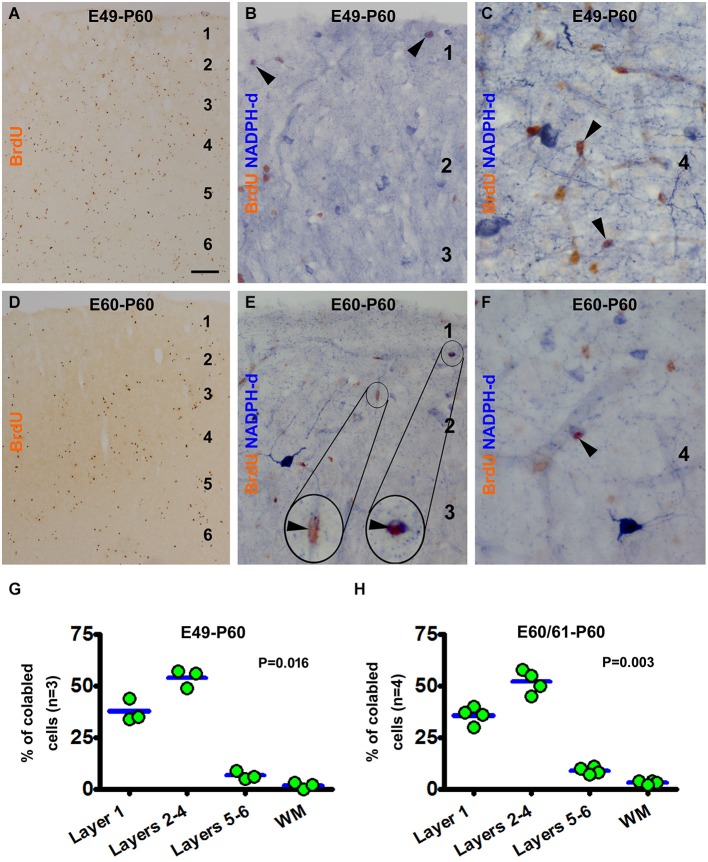
**Examples of images and laminar quantifications for bromodeoxyuridine (BrdU) labeling and colocalization in type II NADPH-d neurons in 2 month-old guinea pigs following prenatal BrdU pulse-chasing**. Shown are the E49-P60 **(A–C,G)** and E60-P60 **(D–F,H)** groups. BrdU immunoreactive cells are present across the cortex without apparent laminar preference in both groups, with noticeably less amount cells in the latter group **(A,D)**. High magnification images illustrate BrdU colabeling in a few type II NADPH-d neurons (arrowheads and inserts) **(B,C,E,F)**, with no colocalization in type I neurons. Over 50% and 30% of the colabeled type II cells occur in layers 2–4 and layer I, respectively for both groups **(G,H)**. Scale bar = 100 μm in **(A)** applying to **(D)**, equivalent to 20 μm for **(B,E)** and 10 μm for **(C,F)**.

**Figure 5:**
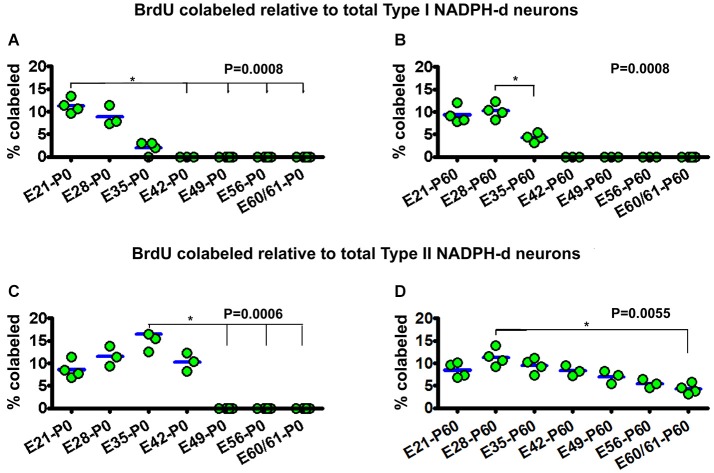
**Dot graphs summarizing the percentages of bromodeoxyuridine (BrdU) labeled relative to the total population of type I (A,B) and type II (C,D) NADPH-d neurons among animal groups examined at postnatal day 0 (P0) (A,C) and P60 (B,D) following BrdU pulse-chasing at different embryonic (E) days (E21, E28, E35, E42, E49, E56 and E60/61)**. Each dot represents the mean from an individual animal. The mean is designated as zero for the animal if no BrdU colocalization in type I or type II NADPH-d neurons can be found by examination of multiple sections. BrdU colocalization in type I NADPH-d neurons can be detected in P0 and P60 animals received BrdU injections at E21, E28 and E35, variably from 2–11.3% group-wise **(A,B)**. BrdU colocalization in type II NADPH-d neurons exists in P0 animal groups received BrdU injections at E21, E28, E35 and E42, variably from 8.6%–16.5% among individual groups. In P60 animals, BrdU colocalization in type II NADPH-d neurons is found in animal groups received BrdU injections at all prenatal time points (i.e., E21, E28, E35, E42, E49, E56 and E60/61), ranging from 4.8–11.3% among individual groups. *P* values represent comparison by Kruskal-Wallis test, with differences (per Dunn’s comparison test) between individual groups marked by lines and asterisks.

A partial colocalization of BrdU and NADPH-d was observed during microscopic examination of the sections from animals surviving 1 and 2 months following prenatal BrdU pulse-chasing. Overall, the pattern and extent of BrdU colocalization in types I and II NADPH-d neurons in 1 month-old animals (*n* = 2 for most groups receiving prenatal BrdU administration, except *n* = 3 for the E49-P30 and E60/60-P30 groups) appeared largely similar to that described above for the P0 groups. Specifically, BrdU localization was found in type I cells in P30 animals received pulse-chasing on E21, E28 and E35, and in type II cells received the chasing on E21, E28, E35 and E42. For simplicity, data from the 1-month old animals are not presented here. In the 2-month-old animals, type I NADPH-d neurons partially co-expressed BrdU in the groups received pulse-chasing by but not later than E35, while a subset of type II NADPH-d neurons exhibited colocalization with BrdU following pulse-chasing at all prenatal time points (Figures 4, 5).

Illustrated as examples, in the E49-P60 group BrdU immunoreactive cells were present across the CP with some cells also in layer 1 and white matter (Figure 4A). BrdU/NADPH-d double-labeled type II, but not type I, cells were found in the cortex predominately in layer 1 and the supragranular layers (Figures 4B,C). Quantitative analysis indicated that 37.7 ± 5.5% of the colabeled cells occurred in layer 1, 54.0 ± 4.4% cells in layers 2–4, 6.7 ± 2.8% in layers 5/6, and 1.7 ± 1.5% in the white matter, with an differential laminar pattern (*P* = 0.016) (Figure 4G). In the E60-P60 group, BrdU immunoreactive cells occurred in all cortical layers and white matter (Figure 4D), noticeably less abundant relative to the E49-P60 group (Figure 4A). BrdU/NADPH-d colabeled cells, all morphologically featured as type II neurons, were present mostly over layer 1 and supragranular layers (Figures 4E,F). Cell count analysis showed that 35.8 ± 3.6% of the colabeled type II neurons were located in layer 1, 52.0 ± 4.9% in layers 2–4, 9 ± 1.6% in layers 5/6, and 3.3 ± 0.8% in the white matter(*P* = 0.003) (Figure 4H).

### Rate of BrdU colabeling in NADPH-d neurons in neonatal and 2 month-old guinea pigs

To determine the relative proportion and timing of type I and type II NADPH-d cells produced at various stages of prenatal development, we calculated the percentage of BrdU colabeled cells against the total population of the cells measured over the parietotemporal neocortical areas. The calculation was based on identification of BrdU reactivity (DAB reaction) in NADPH-d positive neurons. A colabeled cell was defined if there existed at 3 small granules or a large chunk of brown DAB deposit clearly present in the nucleus by high resolution. All colabeled and the NADPH-d single labeled cells present across the entire analyzed image covering layer 1 to the white matter were summed. For type I cells, the judgment was made in some cases with a lowering the contrast of the image if the blue NADPH-d reaction was too intense (which could mask the brown DAB product).

For type I NADPH-d neurons, 11.3 ± 1.6% of the total population were colabeled with BrdU in the E21-P0 group. The colocalization rates were 8.9 ± 2.2% and 2 ± 1.4% for the E28-P0 and E35-P0 groups, respectively. No type I NADPH-d neurons were colabeled for BrdU at P0 following BrdU pulse-chasing at E42, E49, E56 and E60/61 (Figure 5A). There was an overall statistically significant difference between the groups (*P* = 0.001), with Post Test indicating difference for the E21-P0 group relative to E42-P0, E56-P0 and E60/61-P0 groups. In the E21-P60 group, 9.3 ± 1.9% of type I NADPH-d neurons were colabeled with BrdU. In the E28-P60 and E35-P60 groups, 8.9 ± 2.2% and 2 ± 1.4% of type I neurons co-expressed BrdU, respectively. BrdU pulse-chasing at E42, E49, E56 and E60/61 did not yield BrdU colabeling in type I neurons in the 2 month-old offspring (Figure 5B). There was an overall difference between the groups (*P* = 0.001).

For type II NADPH-d neurons, 8.6 ± 2.0% of the total population showed BrdU colabeling in the E21-P0 group, while 11.5 ± 2.2%, 16.5 ± 3.8% and 10.3 ± 2.5% exhibited BrdU immunoreactivity for the E28-P0, E35-P0 and E42-P0 groups, respectively. In contrast, no type II NADPH-d neurons were colabeled for BrdU at P0 following BrdU pulse-chasing at E49, E56 and E60/61 (Figure 5C) (*P* = 0.006, with Post Test indicating difference for the E35-P0 relative to E42-P0 and E60/61-P0 groups). Among the 2-month-old groups, 8.6 ± 1.7% of total type II NADPH-d neurons displayed BrdU immunoreactivity in the E21-P60 group, while 11.3 ± 1.9%, 9.5 ± 1.6% and 8.3 ± 1.2% of the type II cells co-expressed BrdU, respectively, following pulse-chasing at E28, E35 and E42 (Figure 5D). Notably, 7.0 ± 1.4%, 5.5 ± 0.9% and 4.3 ± 1.2% type II cells among the total population were immunoreactive for BrdU following pulse-chasing on E49, E56 and E60/61, respectively (Figure 5D). While there existed an overall difference among the groups (*P* = 0.005), Post Test indicates significant difference between E28-P60 and E60/61-P60 groups) (Figure 5D).

## Discussion

Compared to other species including nonhuman primates, little information about prenatal cortical development and neurogenesis in guinea pigs is currently available. Specifically, the time frame for the genesis of neocortical GABAergic interneuron subgroups remains unknown. In the present study we use BrdU birth-dating and double-labeling methods to explore the ontogenesis of type I and type II NADPH-d reactive interneurons in guinea pig neocortex. Following BrdU injections at selected embryonic time points, incorporation of this exogenous cell division marker can be identified in a subgroup of NADPH-d reactive neurons in postnatal cerebral cortex. Analyses of BrdU/NADPH-d colabeled neurons allow us to gain the information about the timing of overall prenatal cell proliferation relative to cell lamination in postnatal neocortex, duration of the production of type I and type II NADPH-d neurons, and an estimate of time delay for type II neurons being differentiated phenotypically following BrdU incorporation.

### Prenatal cell genesis and lamination in guinea pig neocortex

Mammalian corticogenesis involves an initial formation of the germinal ventricular zone (VZ) and the preplate (PP). The PP contains the early appearing Cajal-Retzius cells and some GABAergic pioneer neurons (Marin-Padilla, 1978; Raedler and Raedler, 1978; Luskin and Shatz, 1985a; Molnár et al., 2006). This is followed by the development of the CP, which splits the PP into the MZ, the future layer 1, and the subplate (SP). The CP is differentiated sequentially into layer 6–2 by the arrival of the radially migrating excitatory pyramidal neurons generated in VZ/SVZ, with later-born neurons bypassing and residing superficially to earlier-born neurons. This cortical genesis framework is established by [3H]-thymidine autoradiographic studies in mouse (Angevine and Sidman, 1961), rat (Bayer and Altman, 1990; Ignacio et al., 1995), cat (Luskin and Shatz, 1985b), ferret (Jackson et al., 1989) and nonhuman primates (Rakic, 1974), and is supported by later cell birth-dating and genetic studies, while more complex pattern of cell proliferation and migration appear to occur in phylogenetically high relative to low mammals (for reviews, see Florio and Huttner, 2014).

Using BrdU birth-dating method the present study extends the first preliminary set of data of prenatal neocortical cell production and lamination in guinea pigs. Thus, BrdU pulse-chasing around the third embryonic week reveals labeled cells largely in layers 3–5 in neonatal and young adult neocortex, while cells generated by the 5th embryonic week are localized predominantly to layer 2. These data are consistent with the general inside-out lamination order of corticogenesis in mammals, representing the formation and final settling down of cortical principal neurons (Florio and Huttner, 2014). However, it should be noted that a substantial population of cells appears to be continuously generated in guinea pig neocortex after the cells destined to layer 2 are born, from the 6th embryonic week to at least until birth. These late born-cells reside across the cortical gray matter without a particular laminar preference. While the precise fates of the late-born cells remain to be characterized, some of them would contribute to the population of type II NADPH-d neurons (to be discussed further).

### Prenatal genesis of type I NADPH-d neurons in guinea pigs

Type I NADPH-d neurons are large in size with long range dendritic and axonal processes capable of modulating cortical neuronal activity in wide areas (Higo et al., 2007; Tamamaki and Tomioka, 2010). In all mammals studied so far these cells are present largely in the subcortical white matter (Estrada and DeFelipe, 1998; Judas et al., 1999), an area related to the early-formed SP (Luskin and Shatz, 1985a; Kostovic and Rakic, 1990; Aboitiz et al., 2005). In the human cerebrum, type I NADPH-d neurons are readily detectable in the subcortical area of the developing neocortex in the first trimester of gestation (Yan et al., 1996b; Yan and Ribak, 1997b; Judas et al., 1999). Thus, it appears that type I NADPH-d cells may be one of the earliest produced or chemically-differentiated neuronal phenotypes in the mammalian cerebrum.

The present BrdU birth-dating study has verified that type I NADPH-d neurons are a group of early-born neurons in guinea pig neocortex. Specifically, these neurons appear to be generated no later than the 5th embryonic week in guinea pigs. In other words, they are produced essentially before the cortical principal neurons destined to layer II are born. It is also of note that type I NADPH-d neurons in the superficial cortical layers, including layer I, are produced during the same embryonic period as with the bulk of cells in the subcortical white matter. This finding indicates that early-born cortical interneurons do not appear to follow the inside-out lamination pattern typical for cortical principal neurons. Given their early-born feature and their final destinations being the subcortical white matter with a few also in layer 1, some of these cells could be produced probably around the time when the cells in the PP are generated.

### Prenatal genesis of type II NADPH-d neurons in guinea pigs

Previous studies have shown that type II NADPH-d neurons are present in the prenatal human cerebral neocortex with a fairly low density (Yan et al., 1996b; Yan and Garey, 1997a; Judas et al., 1999). In contrast, in adult human or monkey neocortex, type II cortical neurons are considerably abundant especially in the supragranular layers (Hashikawa et al., 1994; Barone and Kennedy, 2000; Garbossa et al., 2005; Cruz-Rizzolo et al., 2006). Therefore, one may speculate that type II neurons could belong to a subpopulation of late-born GABAergic interneurons.

The present BrdU birth-chasing study reveals that type II NADPH-d neurons in guinea pig neocortex could be generated during a surprisingly long period of prenatal corticogenesis. Thus, BrdU colabeled type II neurons in neonatal and 1–2 month old guinea pigs are detected following birth-dating as early as the third embryonic to just before birth. Although it remains debated whether there exists constitutive neurogenesis in the adult mammalian neocortex, neurogenesis was described in the adult guinea pig visual cortex a half century ago with the [3H]-thymidine autoradiographic method (Altman and Das, 1967). The finding of type II NADPH-d neurons being produced until birth raises a question as to whether they could be produced in postnatal life.

In the present study we observe that BrdU/NADPH-d colabeled neurons (including type I and type II, primarily the latter due to the difference in population size) produced during E21-E28 tend to distribute somewhat more evenly across the cortical gray matter relative to those generated later, especially after E35. Thus, with the advance of embryonic age, the colabeled neurons become more concentrated (80–90%) in layers 1–4, while very few of these cells (<5%) reside in the white matter. Notably, layer 1 contains a substantial subpopulation of the colabeled neurons following BrdU birth-dating at all of the examined embryonic time points. Interneurons in the mammalian cerebral cortex are reported to predominantly derive from the subpallial ganglionic eminences, including the MGE, LGE and CGE (Lavdas et al., 1999; Wichterle et al., 1999; Ang et al., 2003). However, studies including those from primates have also suggested pallial origins of cortical interneurons, including layer 1 (Soriano et al., 1992; Zecevic and Rakic, 2001; Hevner et al., 2004; Rymar and Sadikot, 2007; Xiong et al., 2010). For instance, pallial production of calretinin neurons during the second trimester may substantially contribute to cortical GABAergic interneuron pool in monkeys (Hladnik et al., 2014). Comparing the laminar distribution of BrdU/NADPH-d type II neurons in different groups with increasing survival duration, it appears that these cells may follow an outside-in migratory pattern. As many type II cells in layer 1 are colabeled following BrdU injections at later embryonic time points (e.g., after E35), cell proliferation leading to interneuron formation clearly occurs in guinea pigs after the basic lamination of the neocortex is established.

### Postmitotic differentiation of type II neurons in guinea pigs

Neurogenesis consists of a continuing process whereby postmitotic cells develop into immature and then mature neurons, with specific markers being expressed at different stages (von Bohlen Und Halbach, 2007). BrdU birth-dating may allow an estimate of the time period during which the labeled postmitotic cells develop and mature into chemically differentiated neurons (Taupin, 2007). Such developmental time period for either the general population or specific subgroups of cortical GABAergic interneurons remains largely unclear to date in most mammals. As discussed above, our results suggest that type I NADPH-d neurons are produced fairly early relative to corticogenesis in guinea pigs. However, as BrdU pulse-chased brains are not examined prenatally in the present study, it is not feasible to estimate how long it would take for type I neurons to become NADPH-d reactive following the cell division that incorporates the injected BrdU.

The data obtained in the present study however allow a rough prediction that type II neurons would likely take more than 1 month following BrdU incorporation to become NADPH-d reactive. This estimate is based on whether and when BrdU/NADPH-d colabeled type II neurons are detected in postnatal neocortex in the groups received pulse-chasing at different embryonic time points. We find that no BrdU/NADPH-d colabeled type II neurons are detectable in neonatal and 1 month-old guinea pigs with BrdU injected on and after E49. In contrast, BrdU/NADPH-d colabeled type II neurons are present in 2 month-old animals following BrdU injections at E49, E56 and E60/61. Thus, the finding of BrdU/NADPH-d colocalization in type II neurons in the E49-P60 group, but not in the E49-P0 group, would implicate that type II neurons become NADPH-d positive between approximately 40 and 70 days following mitosis (roughly counting guinea pig pregnant time as ~60 days here). By the same token, the lack of BrdU/NADPH-d colocalization in type II neurons in neonatal and 1 month-old, but the presence of which in 2 month-old, animals following BrdU injections on E60/61 would suggest that there is a delay between 30 and 60 days for postmitotic precursors to be detected by NADPH-d histochemistry.

In summary, the present study demonstrates that type I nitrinergic interneurons in guinea pig cerebral neocortex are generated during embryonic day 21–35 (potentially earlier). Type II NADPH-d neurons may be generated over a wide prenatal time window, from embryonic day 21 until birth. The bulk of cells to build the CP are produced no later than the fifth embryonic week. However, considerable amounts of cortical cells continue to be generated until birth. Overall, these results may implicate that the formation of type I NADPH-d neurons could be affected by early embryonic insults, while certain late prenatal and postnatal factors could impact the development of type II nitrinergic neuronal population in mammalian cerebral cortex.

## Conflict of interest statement

The authors declare that the research was conducted in the absence of any commercial or financial relationships that could be construed as a potential conflict of interest.

## References

[B1] Aboitiz F. Montiel J. García R. R. (2005). Ancestry of the mammalian preplate and its derivatives: evolutionary relicts or embryonic adaptations? Rev. Neurosci. 16, 359–376. 10.1515/revneuro.2005.16.4.359 16519011

[B2] Altman J. Das G. D. (1967). Postnatal neurogenesis in the guinea-pig. Nature 214, 1098–1101. 10.1038/2141098a0 6053066

[B3] Ang E. S. Jr. Haydar T. F. Gluncic V. Rakic P. (2003). Four-dimensional migratory coordinates of GABAergic interneurons in the developing mouse cortex. J. Neurosci. 23, 5805–5815. 12843285 10.1523/JNEUROSCI.23-13-05805.2003 PMC6741259

[B4] Angevine J. B. Jr. Sidman R. L. (1961). Autoradiographic study of cell migration during histogenesis of cerebral cortex in the mouse. Nature 192, 766–768. 10.1038/192766b0 17533671

[B5] Aoki C. Fenstemaker S. Lubin M. Go C. G. (1993). Nitric oxide synthase in the visual cortex of monocular monkeys as revealed by light and electron microscopic immunocytochemistry. Brain Res. 620, 97–113. 10.1016/0006-8993(93)90275-r 7691382

[B6] Aquilano K. Baldelli S. Rotilio G. Ciriolo M. R. (2008). Role of nitric oxide synthases in Parkinson’s disease: a review on the antioxidant and anti-inflammatory activity of polyphenols. Neurochem. Res. 33, 2416–2426. 10.1007/s11064-008-9697-6 18415676

[B7] Barone P. Kennedy H. (2000). Non-uniformity of neocortex: areal heterogeneity of NADPH-diaphorase reactive neurons in adult macaque monkeys. Cereb. Cortex 10, 160–174. 10.1093/cercor/10.2.160 10667984

[B8] Bayer S. A. Altman J. (1990). Development of layer I and the subplate in the rat neocortex. Exp. Neurol. 107, 48–62. 10.1016/0014-4886(90)90062-w 2295319

[B9] Belvisi M. G. Ward J. K. Mitchell J. A. Barnes P. J. (1995). Nitric oxide as a neurotransmitter in human airways. Arch. Int. Pharmacodyn. Ther. 329, 97–110. 7639623

[B10] Bonfanti L. Nacher J. (2012). New scenarios for neuronal structural plasticity in non-neurogenic brain parenchyma: the case of cortical layer II immature neurons. Prog. Neurobiol. 98, 1–15. 10.1016/j.pneurobio.2012.05.002 22609484

[B11] Bredt D. S. Hwang P. M. Snyder S. H. (1990). Localization of nitric oxide synthase indicating a neural role for nitric oxide. Nature 347, 768–770. 10.1038/347768a0 1700301

[B13] Cahalane D. J. Charvet C. J. Finlay B. L. (2014). Modeling local and cross-species neuron number variations in the cerebral cortex as arising from a common mechanism. Proc. Natl. Acad. Sci. U S A 111, 17642–17647. 10.1073/pnas.1409271111 25422426 PMC4267349

[B14] Cai Y. Xiong K. Chu Y. Luo D. W. Luo X. G. Yuan X. Y.. (2009). Doublecortin expression in adult cat and primate cerebral cortex relates to immature neurons that develop into GABAergic subgroups. Exp. Neurol. 216, 342–356. 10.1016/j.expneurol.2008.12.008 19166833 PMC2902881

[B15] Ceccatelli S. (1997). Expression and plasticity of NO synthase in the neuroendocrine system. Brain Res. Bull. 44, 533–538. 10.1016/s0361-9230(97)00239-6 9370221

[B16] Cherian L. Hlatky R. Robertson C. S. (2004). Nitric oxide in traumatic brain injury. Brain Pathol. 14, 195–201. 10.1111/j.1750-3639.2004.tb00053.x 15193032 PMC8095951

[B17] Colvin S. M. Kwan K. Y. (2014). Dysregulated nitric oxide signaling as a candidate mechanism of fragile X syndrome and other neuropsychiatric disorders. Front. Genet. 5:239. 10.3389/fgene.2014.00239 25101118 PMC4105824

[B18] Cruz-Rizzolo R. J. Horta-Júnior J. A. Bittencourt J. C. Ervolino E. de Oliveira J. A. Casatti C. A. (2006). Distribution of NADPH-diaphorase-positive neurons in the prefrontal cortex of the Cebus monkey. Brain Res. 1083, 118–133. 10.1016/j.brainres.2006.01.098 16530735

[B19] Cudeiro J. Rivadulla C. Grieve K. L. (2000). A possible role for nitric oxide at the sleep/wake interface. Sleep 23, 829–835. 11007450

[B20] D’Alessio L. López-Costa J. J. Konopka H. Consalvo D. Seoane E. López M. E.. (2007). NADPH diaphorase reactive neurons in temporal lobe cortex of patients with intractable epilepsy and hippocampal sclerosis. Epilepsy Res. 74, 228–231. 10.1016/j.eplepsyres.2007.02.007 17412562

[B21] Dawson T. M. Bredt D. S. Fotuhi M. Hwang P. M. Snyder S. H. (1991). Nitric oxide synthase and neuronal NADPH diaphorase are identical in brain and peripheral tissues. Proc. Natl. Acad. Sci. U S A 88, 7797–7801. 10.1073/pnas.88.17.7797 1715581 PMC52390

[B22] Estrada C. DeFelipe J. (1998). Nitric oxide-producing neurons in the neocortex: morphological and functional relationship with intraparenchymal microvasculature. Cereb. Cortex 8, 193–203. 10.1093/cercor/8.3.193 9617914

[B23] Florio M. Huttner W. B. (2014). Neural progenitors, neurogenesis and the evolution of the neocortex. Development 141, 2182–2194. 10.1242/dev.090571 24866113

[B24] Freire M. A. Faber J. Picanço-Diniz C. W. Franca J. G. Pereira A. (2012). Morphometric variability of nicotinamide adenine dinucleotide phosphate diaphorase neurons in the primary sensory areas of the rat. Neuroscience 205, 140–153. 10.1016/j.neuroscience.2011.12.029 22226695

[B25] Gądek-Michalska A. Tadeusz J. Rachwalska P. Bugajski J. (2013). Cytokines, prostaglandins and nitric oxide in the regulation of stress-response systems. Pharmacol. Rep. 65, 1655–1662. 10.1016/s1734-1140(13)71527-5 24553014

[B26] Garbossa D. Fontanella M. Tomasi S. Ducati A. Vercelli A. (2005). Differential distribution of NADPH-diaphorase histochemistry in human cerebral cortex. Brain Res. 1034, 1–10. 10.1016/j.brainres.2004.10.049 15713254

[B27] Garrel G. Lozach A. Bachir L. K. Laverriere J. N. Counis R. (2002). Pituitary adenylate cyclase-activating polypeptide stimulates nitric-oxide synthase type I expression and potentiates the cGMP response to gonadotropin-releasing hormone of rat pituitary gonadotrophs. J. Biol. Chem. 277, 46391–46401. 10.1074/jbc.m203763200 12244042

[B28] Gautier-Sauvigné S. Colas D. Parmantier P. Clement P. Gharib A. Sarda N.. (2005). Nitric oxide and sleep. Sleep Med. Rev. 9, 101–113. 10.1016/j.smrv.2004.07.004 15737789

[B29] Gerashchenko D. Wisor J. P. Burns D. Reh R. K. Shiromani P. J. Sakurai T.. (2008). Identification of a population of sleep-active cerebral cortex neurons. Proc. Natl. Acad. Sci. U S A 105, 10227–10232. 10.1073/pnas.0803125105 18645184 PMC2481371

[B30] Gibbs S. M. (2003). Regulation of neuronal proliferation and differentiation by nitric oxide. Mol. Neurobiol. 27, 107–120. 10.1385/mn:27:2:107 12777682

[B31] Givalois L. Li S. Pelletier G. (2002). Central nitric oxide regulation of the hypothalamic-pituitary-adrenocortical axis in adult male rats. Brain Res. Mol. Brain Res. 102, 1–8. 10.1016/s0169-328x(02)00218-8 12191488

[B32] Greene R. W. (2013). Role for neuronal nitric oxide synthase in sleep homeostasis and arousal. Proc. Natl. Acad. Sci. U S A 110, 19982–19983. 10.1073/pnas.1319863110 24284175 PMC3864275

[B33] Guix F. X. Uribesalgo I. Coma M. Muñoz F. J. (2005). The physiology and pathophysiology of nitric oxide in the brain. Prog. Neurobiol. 76, 126–152. 10.1016/j.pneurobio.2005.06.001 16115721

[B34] Hardingham N. Dachtler J. Fox K. (2013). The role of nitric oxide in pre-synaptic plasticity and homeostasis. Front. Cell. Neurosci. 7:190. 10.3389/fncel.2013.00190 24198758 PMC3813972

[B35] Hashikawa T. Leggio M. G. Hattori R. Yui Y. (1994). Nitric oxide synthase immunoreactivity colocalized with NADPH-diaphorase histochemistry in monkey cerebral cortex. Brain Res. 641, 341–349. 10.1016/0006-8993(94)90164-3 7516813

[B36] Herculano-Houzel S. (2007). Encephalization, neuronal excess and neuronal index in rodents. Anat. Rec. (Hoboken) 290, 1280–1287. 10.1002/ar.20598 17847061

[B37] Hevner R. F. Daza R. A. Englund C. Kohtz J. Fink A. (2004). Postnatal shifts of interneuron position in the neocortex of normal and reeler mice: evidence for inward radial migration. Neuroscience 124, 605–618. 10.1016/j.neuroscience.2003.11.033 14980731

[B38] Higo S. Udaka N. Tamamaki N. (2007). Long-range GABAergic projection neurons in the cat neocortex. J. Comp. Neurol. 503, 421–431. 10.1002/cne.21395 17503478

[B39] Hladnik A. Džaja D. Darmopil S. Jovanov-Milošević N. Petanjek Z. (2014). Spatio-temporal extension in site of origin for cortical calretinin neurons in primates. Front. Neuroanat. 8:50. 10.3389/fnana.2014.00050 25018702 PMC4072090

[B40] Hope B. T. Michael G. J. Knigge K. M. Vincent S. R. (1991). Neuronal NADPH diaphorase is a nitric oxide synthase. Proc. Natl. Acad. Sci. U S A 88, 2811–2814. 10.1073/pnas.88.7.2811 1707173 PMC51329

[B41] Ignacio M. P. Kimm E. J. Kageyama G. H. Yu J. Robertson R. T. (1995). Postnatal migration of neurons and formation of laminae in rat cerebral cortex. Anat. Embryol. (Berl) 191, 89–100. 10.1007/bf00186782 7726396

[B42] Jackson C. A. Peduzzi J. D. Hickey T. L. (1989). Visual cortex development in the ferret. I. Genesis and migration of visual cortical neurons. J. Neurosci. 9, 1242–1253. 2703875 10.1523/JNEUROSCI.09-04-01242.1989 PMC6569864

[B43] Jaglin X. H. Hjerling-Leffler J. Fishell G. Batista-Brito R. (2012). The origin of neocortical nitric oxide synthase-expressing inhibitory neurons. Front. Neural Circuits 6:44. 10.3389/fncir.2012.00044 22787442 PMC3391688

[B44] Judas M. Sestan N. Kostović I. (1999). Nitrinergic neurons in the developing and adult human telencephalon: transient and permanent patterns of expression in comparison to other mammals. Microsc. Res. Tech. 45, 401–419. 10.1002/(sici)1097-0029(19990615)45:6<401::aid-jemt7>3.3.co;2-h 10402267

[B46] Kostovic I. Rakic P. (1990). Developmental history of the transient subplate zone in the visual and somatosensory cortex of the macaque monkey and human brain. J. Comp. Neurol. 297, 441–470. 10.1002/cne.902970309 2398142

[B47] Lavdas A. A. Grigoriou M. Pachnis V. Parnavelas J. G. (1999). The medial ganglionic eminence gives rise to a population of early neurons in the developing cerebral cortex. J. Neurosci. 19, 7881–7888. 10479690 10.1523/JNEUROSCI.19-18-07881.1999 PMC6782477

[B48] Leigh P. N. Connick J. H. Stone T. W. (1990). Distribution of NADPH-diaphorase positive cells in the rat brain. Comp. Biochem. Physiol. C 97, 259–264. 10.1016/0742-8413(90)90138-y 1982868

[B49] Luskin M. B. Shatz C. J. (1985a). Studies of the earliest generated cells of the cat’s visual cortex: cogeneration of subplate and marginal zones. J. Neurosci. 5, 1062–1075. 3981242 10.1523/JNEUROSCI.05-04-01062.1985 PMC6565007

[B50] Luskin M. B. Shatz C. J. (1985b). Neurogenesis of the cat’s primary visual cortex. J. Comp. Neurol. 242, 611–631. 10.1002/cne.902420409 4086673

[B51] Lüth H. J. Hedlich A. Hilbig H. Winkelmann E. Mayer B. (1994). Morphological analyses of NADPH-diaphorase/nitric oxide synthase positive structures in human visual cortex. J. Neurocytol. 23, 770–782. 10.1007/bf01268089 7534823

[B52] Magno L. Oliveira M. G. Mucha M. Rubin A. N. Kessaris N. (2012). Multiple embryonic origins of nitric oxide synthase-expressing GABAergic neurons of the neocortex. Front. Neural Circuits 6:65. 10.3389/fncir.2012.00065 23015780 PMC3449337

[B53] Majlessi N. Choopani S. Bozorgmehr T. Azizi Z. (2008). Involvement of hippocampal nitric oxide in spatial learning in the rat. Neurobiol. Learn. Mem. 90, 413–419. 10.1016/j.nlm.2008.04.010 18508394

[B54] Marin-Padilla M. (1978). Dual origin of the mammalian neocortex and evolution of the cortical plate. Anat. Embryol. (Berl) 152, 109–126. 10.1007/bf00315920 637312

[B55] Matarredona E. R. Murillo-Carretero M. Moreno-López B. Estrada C. (2005). Role of nitric oxide in subventricular zone neurogenesis. Brain Res. Brain Res. Rev. 49, 355–366. 10.1016/j.brainresrev.2005.01.001 16111562

[B56] McCann S. M. Mastronardi C. de Laurentiis A. Rettori V. (2005). The nitric oxide theory of aging revisited. Ann. N Y Acad. Sci. 1057, 64–84. 10.1196/annals.1356.064 16399888

[B57] McLeod T. M. López-Figueroa A. L. López-Figueroa M. O. (2001). Nitric oxide, stress and depression. Psychopharmacol. Bull. 35, 24–41. 12397868

[B58] Melikian N. Seddon M. D. Casadei B. Chowienczyk P. J. Shah A. M. (2009). Neuronal nitric oxide synthase and human vascular regulation. Trends Cardiovasc. Med. 19, 256–262. 10.1016/j.tcm.2010.02.007 20447567 PMC2984617

[B59] Molnár Z. Métin C. Stoykova A. Tarabykin V. Price D. J. Francis F.. (2006). Comparative aspects of cerebral cortical development. Eur. J. Neurosci. 23, 921–934. 10.1111/j.1460-9568.2006.04611.x 16519657 PMC1931431

[B60] Nogueira-Campos A. A. Finamore D. M. Imbiriba L. A. Houzel J. C. Franca J. G. (2012). Distribution and morphology of nitrergic neurons across functional domains of the rat primary somatosensory cortex. Front. Neural Circuits 6:57. 10.3389/fncir.2012.00057 23133407 PMC3490138

[B61] Paul V. Ekambaram P. (2011). Involvement of nitric oxide in learning & memory processes. Indian J. Med. Res. 133, 471–478. 21623030 PMC3121276

[B62] Raedler E. Raedler A. (1978). Autoradiographic study of early neurogenesis in rat neocortex. Anat. Embryol. (Berl) 154, 267–284. 10.1007/bf00345657 707818

[B63] Rakic P. (1974). Neurons in rhesus monkey visual cortex: systematic relation between time of origin and eventual disposition. Science 183, 425–427. 10.1126/science.183.4123.425 4203022

[B64] Reif A. Herterich S. Strobel A. Ehlis A. C. Saur D. Jacob C. P.. (2006). A neuronal nitric oxide synthase (NOS-I) haplotype associated with schizophrenia modifies prefrontal cortex function. Mol. Psychiatry 11, 286–300. 10.1038/sj.mp.4001779 16389274

[B65] Rice F. L. Gomez C. Barstow C. Burnet A. Sands P. (1985). A comparative analysis of the development of the primary somatosensory cortex: interspecies similarities during barrel and laminar development. J. Comp. Neurol. 236, 477–495. 10.1002/cne.902360405 4056099

[B66] Riedel W. (2000). Role of nitric oxide in the control of the hypothalamic-pituitary-adrenocortical axis. Z. Rheumatol. 59(Suppl 2), II36–II42. 10.1007/s003930070016 11155802

[B67] Rodrigo J. Springall D. R. Uttenthal O. Bentura M. L. Abadia-Molina F. Riveros-Moreno V.. (1994). Localization of nitric oxide synthase in the adult rat brain. Philos. Trans. R. Soc. Lond. B Biol. Sci. 345, 175–221. 10.1098/rstb.1994.0096 7526408

[B68] Rymar V. V. Sadikot A. F. (2007). Laminar fate of cortical GABAergic interneurons is dependent on both birthdate and phenotype. J. Comp. Neurol. 501, 369–380. 10.1002/cne.21250 17245711

[B69] Salemme E. Diano S. Maharajan P. Maharajan V. (1996). Nitric oxide, a neuronal messenger. Its role in the hippocampus neuronal plasticity. Riv. Biol. 89, 87–107. 9122583

[B70] Schüz A. (1981). Prenatal development and postnatal changes in the guinea pig cortex: microscopic evaluation of a natural deprivation experiment. I. Prenatal development. J. Hirnforsch. 22, 93–111. 7240730

[B71] Soriano E. Del Rio J. A. Ferrer I. Auladell C. De Lecea L. Alcantara S. (1992). Late appearance of parvalbumin-immunoreactive neurons in the rodent cerebral cortex does not follow an ‘inside-out’ sequence. Neurosci. Lett. 142, 147–150. 10.1016/0304-3940(92)90360-j 1454208

[B72] Sunico C. R. Portillo F. González-Forero D. Moreno-López B. (2005). Nitric-oxide-directed synaptic remodeling in the adult mammal CNS. J. Neurosci. 25, 1448–1458. 10.1523/jneurosci.4600-04.2005 15703399 PMC6725993

[B73] Tamamaki N. Tomioka R. (2010). Long-range GABAergic connections distributed throughout the neocortex and their possible function. Front. Neurosci. 4:202. 10.3389/fnins.2010.00202 21151790 PMC3000116

[B74] Taupin P. (2007). BrdU immunohistochemistry for studying adult neurogenesis: paradigms, pitfalls, limitations and validation. Brain Res. Rev. 53, 198–214. 10.1016/j.brainresrev.2006.08.002 17020783

[B75] Toda N. Ayajiki K. Okamura T. (2009). Cerebral blood flow regulation by nitric oxide: recent advances. Pharmacol. Rev. 61, 62–97. 10.1124/pr.108.000547 19293146

[B76] Togo T. Katsuse O. Iseki E. (2004). Nitric oxide pathways in Alzheimer’s disease and other neurodegenerative dementias. Neurol. Res. 26, 563–566. 10.1179/016164104225016236 15265275

[B77] Valtschanoff J. G. Weinberg R. J. Kharazia V. N. Schmidt H. H. Nakane M. Rustioni A. (1993). Neurons in rat cerebral cortex that synthesize nitric oxide: NADPH diaphorase histochemistry, NOS immunocytochemistry and colocalization with GABA. Neurosci. Lett. 157, 157–161. 10.1016/0304-3940(93)90726-2 7694193

[B90] Vincent S. R. Kimura H. (1992). Histochemical mapping of nitric oxide synthase in the rat brain. Neuroscience 46, 755–784. 10.1016/0306-4522(92)90184-4 1371855

[B78] von Bohlen Und Halbach O. (2007). Immunohistological markers for staging neurogenesis in adult hippocampus. Cell Tissue Res. 329, 409–420. 10.1007/s00441-007-0432-4 17541643

[B79] Weber H. Klamer D. Freudenberg F. Kittel-Schneider S. Rivero O. Scholz C. J.. (2014). The genetic contribution of the NO system at the glutamatergic post-synapse to schizophrenia: further evidence and meta-analysis. Eur. Neuropsychopharmacol. 24, 65–85. 10.1016/j.euroneuro.2013.09.005 24220657

[B80] Wichterle H. Garcia-Verdugo J. M. Herrera D. G. Alvarez-Buylla A. (1999). Young neurons from medial ganglionic eminence disperse in adult and embryonic brain. Nat. Neurosci. 2, 461–466. 10.1038/8131 10321251

[B91] Wultsch T. Chourbaji S. Fritzen S. Kittel S. Grünblatt E. Gerlach M.. (2007). Behavioural and expressional phenotyping of nitric oxide synthase-I knockdown animals. J. Neural Transm. Suppl. 72(Suppl), 69–85. 10.1007/978-3-211-73574-9_10 17982880

[B81] Xiong K. Cai Y. Zhang X. M. Huang J. F. Liu Z. Y. Fu G. M.. (2010). Layer I as a putative neurogenic niche in young adult guinea pig cerebrum. Mol. Cell. Neurosci. 45, 180–191. 10.1016/j.mcn.2010.06.009 20599617 PMC2923265

[B82] Xiong K. Luo D. W. Patrylo P. R. Luo X. G. Struble R. G. Clough R. W.. (2008). Doublecortin-expressing cells are present in layer II across the adult guinea pig cerebral cortex: partial colocalization with mature interneuron markers. Exp. Neurol. 211, 271–282. 10.1016/j.expneurol.2008.02.003 18378231 PMC2994188

[B83] Yan X. X. Garey L. J. (1997a). Morphological diversity of nitric oxide synthesising neurons in mammalian cerebral cortex. J. Hirnforsch. 38, 165–172. 9176729

[B84] Yan X. X. Garey L. J. Jen L. S. (1994). Development of NADPH-diaphorase activity in the rat neocortex. Brain Res. Dev. Brain Res. 79, 29–38. 10.1016/0165-3806(94)90046-9 8070062

[B85] Yan X. X. Garey L. J. Jen L. S. (1996b). Prenatal development of NADPH-diaphorase-reactive neurons in human frontal cortex. Cereb. Cortex 6, 737–745. 10.1093/cercor/6.5.737 8921208

[B86] Yan X. X. Jen L. S. Garey L. J. (1996a). NADPH-diaphorase-positive neurons in primate cerebral cortex colocalize with GABA and calcium-binding proteins. Cereb. Cortex 6, 524–529. 10.1093/cercor/6.3.524 8670678

[B87] Yan X. X. Ribak C. E. (1997b). Prenatal development of nicotinamide adenine dinucleotide phosphate-diaphorase activity in the human hippocampal formation. Hippocampus 7, 215–231. 10.1002/(SICI)1098-1063(1997)7:2%3C215::AID-HIPO8%3E3.0.CO;2-L 9136051

[B88] Zecevic N. Rakic P. (2001). Development of layer I neurons in the primate cerebral cortex. J. Neurosci. 21, 5607–5619. 11466432 10.1523/JNEUROSCI.21-15-05607.2001 PMC6762645

[B89] Zimmermann M. (2001). Pathobiology of neuropathic pain. Eur. J. Pharmacol. 429, 23–37. 10.1016/S0014-2999(01)01303-6 11698024

